# Clinical classification systems and long-term outcome in mid- and late-stage Parkinson’s disease

**DOI:** 10.1038/s41531-021-00208-4

**Published:** 2021-08-02

**Authors:** Emil Ygland Rödström, Andreas Puschmann

**Affiliations:** Lund University, Skåne University Hospital, Department of Clinical Sciences Lund, Neurology, Lund, Sweden

**Keywords:** Parkinson's disease, Dementia, Parkinson's disease, Dementia, Prognostic markers

## Abstract

Parkinson’s disease shows a heterogeneous course and different clinical subtyping systems have been described. To compare the capabilities of two clinical classification systems, motor-phenotypes, and a simplified clinical motor-nonmotor subtyping system, a cohort was included at mean 7.9 ± 5.3 years of disease duration, classified using both clinical systems, and reexamined and reclassified at the end of an observation period. Time-points were retrospectively extracted for five major disease milestones: death, dementia, Hoehn and Yahr stage 5, nursing home living, and walking aid use. Eighty-nine patients were observed for 8.1 ± 2.7 years after inclusion. Dementia developed in 32.9% of the patients and 36.0–67.4% reached the other milestones. Motor-phenotypes were unable to stratify risks during this period, but the worst compared with the more favorable groups in the motor-nonmotor system conveyed hazard ratios between 2.6 and 63.6 for all milestones. A clear separation of risks for dying, living at the nursing home, and reaching motor end-stage was also shown when using only postural instability and gait disorder symptoms, without weighing them against the severity of the tremor. At reexamination, 29.4% and 64.7% of patients had changed classification groups in the motor-phenotype and motor-nonmotor systems, respectively. The motor-nonmotor system thus stratified risks of reaching crucial outcomes in mid–late Parkinson’s disease far better than the well-studied motor-phenotypes. Removing the tremor aspect of motor-phenotypes clearly improved this system, however. Classifications in both systems became unstable over time. The simplification of the motor-nonmotor system was easily applicable and showed potential as a prognostic marker during a large part of Parkinson’s disease.

## Introduction

In later stages of Parkinson’s disease (PD), some patients develop dementia or permanently need to use a wheelchair (Hoehn and Yahr stage 5; HY5), whereas others never become as severely affected^1^. Time and pattern of progression to such PD end-stages is heterogeneous^2^ and various classification systems have been developed, trying to elucidate these differences^3–11^.

One frequently used classification system, motor-phenotypes, is based on the predominant motor findings in PD^4^. However, nonmotor symptoms are found to hold prognostic information^12–15^ that are crucial for accurate PD prognostication^5,16^ and have been successfully implemented in several PD classifications^5–9^. Unfortunately, the data-driven approaches commonly used can make replication difficult^17^, and identifying scales and measures that are easily applied in a normal out-patient appointment, and also hold high reproducibility between examiners and patients from different ethnicities, can be challenging^18^. Recently, a study constructed an algorithm for a new clinical subtype classification system, based on cluster analysis of both motor and nonmotor findings^3^. Patients of the different groups were shown to vary in cerebrospinal fluid biomarkers^3^ and clinically and radiologically evaluated disease progression^19–21^. Improved clinical feasibility and confirmation of this system with population-based inclusion and longer time of observation have been requested^22^. The prognostic capabilities of these subtypes have been assessed in two other longitudinal PD cohorts, grouping patients close to diagnosis^19–21^, but, to the best of our knowledge, the long-term efficacy of this system when applied in mid-stage PD has not yet been examined. In this study, we adapted the motor-nonmotor subtyping system to facilitate clinical use and applied both this system and motor-phenotypes in patients with mid and late stages of PD from a cohort with long follow-up, comparing risks for reaching relevant PD milestones.

## Results

### Demographics

Baseline examination was performed at 7.9 ± 5.3 (mean ± SD) years after disease onset in 89 patients with HY-stage 2.8 ± 1.1. Follow-up data covering the following 8.1 ± 2.7 years was extracted from medical records, more specifically until 16.0 ± 5.4 total years of disease duration and 75.7 ± 8.0 years of age. In the most benign groups in each system (mild-motor-predominant and tremor-dominant), there were fewer patients that reached the assessed milestones, but they also had younger-onset ages, a higher proportion of women, and shorter disease duration (Table 1).

**Table 1:** Demographics and follow-up data.

Data	Total	Missing^a^	TD	U	PIGD	MMP	IM	DM
Number of patients (*n*)	89	n/r	16	14	59	35	33	21
Age of onset (yrs)	59.7 ± 9.2	0	57.7 ± 8.9	58.7 ± 10.3	60.4 ± 9.1	58.8 ± 10.0	59.1 ± 9.1	62.1 ± 7.7
Men (*n*)	54 (60.7%)	0	8 (50.0%)	8 (57.1%)	38 (64.4%)	19 (54.3%)	22 (66.7%)	13 (61.9%)
Duration at baseline	7.9 ± 5.3	0	6.7 ± 3.9	8.0 ± 5.8	8.2 ± 5.5	6.1 ± 4.0	7.4 ± 5.4	11.7 ± 5.2
Age at baseline (yrs)	67.6 ± 9.1	0	64.4 ± 8.9	66.8 ± 8.3	68.6 ± 9.2	64.9 ± 9.4	66.5 ± 8.2	73.8 ± 7.0
RBD at baseline (*n*)	33 (41.3%)	9 (10.1%)	4 (26.7%)	6 (50.0%)	23 (43.4%)	0	23 (76.7%)	10 (55.6%)
Hallucinations at baseline	28 (31.5%)	0	1 (6.3%)	5 (35.7%)	22 (37.3%)	0	11 (33.3%)	17 (81.0%)
NMSQ score at baseline	9.8 ± 4.9	7 (7.9%)	7.5 ± 3.4	10.3 ± 5.3	10.3 ± 5.0	6.4 ± 3.5	10.8 ± 4.3	13.6 ± 4.0
Imputation in UPDRS (*n*)	28 (31.5%)	n/r	6 (37.5%)	4 (28.6%)	18 (30.5%)	12 (34.3%)	9 (27.3%)	7 (33.3%)
Regularly used walker before baseline (*n*)	14 (15.7%)	3 (3.4%)^b^	0	2 (14.3%)	12 (20.3%)	0	2 (6.1%)	12 (57.1%)
Moved to nursing home before baseline (*n*)	5 (5.6%)	0	0	0	5 (8.5%)	0	0	5 (23.81%)
Progressed to HY5 before baseline (*n*)	1 (1.1%)	0	0	0	1 (1.7%)	0	0	1 (4.8%)
Developed dementia before baseline (*n*)	7 (7.9%)	7 (7.9%)^b^	0	1 (7.1%)	6 (10.2%)	0	0	7 (33.3%)
**Follow-up data**								
Years followed since baseline	8.1 ± 2.7	0	9.2 ± 1.9	9.5 ± 1.4	7.5 ± 2.9	9.4 ± 1.8	8.0 ± 2.7	6.1 ± 2.8
Disease duration from onset (yrs)	16.0 ± 5.4	0	15.9 ± 4.7	17.5 ± 6.4	15.7 ± 5.4	15.5 ± 4.3	15.4 ± 5.9	17.9 ± 6.0
Age at end of study or at death (yrs)	75.7 ± 8.0	0	73.6 ± 8.9	76.2 ± 7.2	76.1 ± 7.9	74.2 ± 8.3	74.5 ± 8.0	80.0 ± 5.8
Died (*n*)	37 (41.6%)	0	3 (18.8%)	4 (28.6%)	30 (50.8%)	10 (28.6%)	11 (33.3%)	16 (76.2%)
Age at death (yrs)	80.0 ± 5.9	0	80.7 ± 7.2	83.0 ± 2.0	79.5 ± 6.2	81.3 ± 6.1	76.8 ± 5.3	81.4 ± 5.7
Regularly used walker (*n*)	58 (67.4%)	3 (3.4%)^a^	8 (50.0%)	9 (64.3%)	41 (73.2%)	20 (57.1%)	20 (62.5%)	18 (94.7%)
Progressed to HY5 (*n*)	32 (36.0%)	0	3 (18.8%)	4 (28.6%)	25 (42.4%)	7 (20.0%)	10 (30.3%)	15 (71.4%)
Moved to nursing home (*n*)	38 (42.7%)	0	4 (25.0%)	5 (35.7%)	29 (49.2%)	10 (28.6%)	11 (33.3%)	17 (81%)
Dementia (*n*)	27 (32.9%)	7 (7.9%)^a^	2 (13.3%)	3 (25.0%)	22 (40.0%)	6 (19.4%)	8 (25.8%)	13 (65%)
Participation in clinical reexamination (*n*)	34 (38.2%)	0	9 (56.3%)	4 (28.6%)	21 (35.6%)	17 (48.6%)	15 (45.5%)	2 (9.5%)

Cohort demographics and variables included in the classification systems for the whole cohort and subsets. Results are shown in mean ± SD of scores or years (yrs) or in absolute nr (percent of available data for the subset) indicated by (*n*).

^a^Number of patients with missing data (% of the whole cohort).

^b^Individuals for whom the use of walker or dementia could not be unambiguously extracted from available data.

*HY5* Hoehn & Yahr stage 5, *RBD* symptoms suggestive of Rem-sleep behavioral disorder, *NMSQ* nonmotor symptom questionnaire, *TD* Tremor-dominant motor-phenotype, *U* Undetermined motor-phenotype, *PIGD* postural stability and gait disorder motor-phenotype, *MMP* mild-motor-predominant subtype, *IM* Intermediate subtype, *DM* diffuse-malignant subtype.

### Risks of reaching disease milestones

**Fig. 1:**
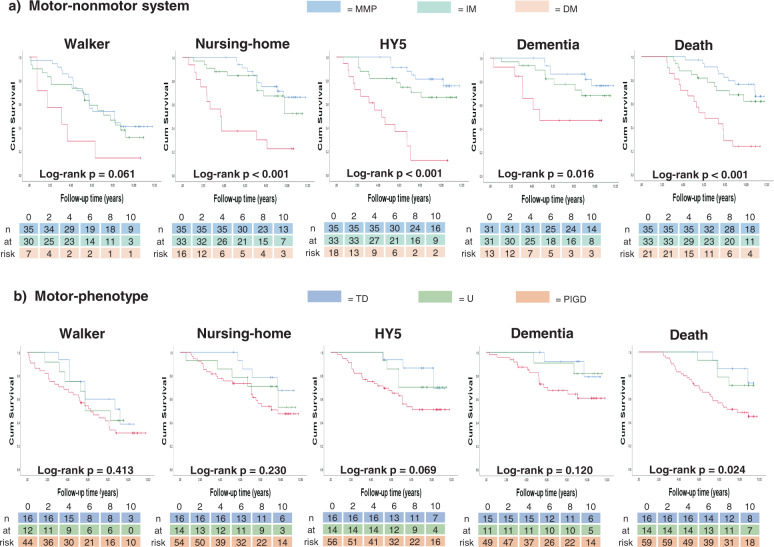
Kaplan–Meier survival curves for reaching disease milestones. Number of cases entering intervals below each graph. **a** Motor-nonmotor subtype classification system outcomes as indicated; **b** Motor-phenotype system outcomes as indicated. MMP Mild-motor-predominant subtype, IM Intermediate subtype, DM Diffuse-malignant subtype, HY5 Hoehn and Yahr stage 5, TD Tremor-dominant motor-phenotype, U Undetermined motor-phenotype, PIGD Postural stability and gait disorder motor-phenotype.

Risks of reaching the five disease milestones differed between the groups of both systems in an ordered fashion (Kaplan–Meier curves, Fig. 1).

In the motor-nonmotor system, log-rank tests showed significant differences in risks between the groups for all outcomes except walker usage. Compared with mild-motor-predominant patients, the diffuse-malignant patients had significantly increased risks for all outcomes, with and without adjustments (Table 2). Hazard ratios (HRs) showed 4.2 (CI 1.2–14.9) times the risk of dementia development in diffuse-malignant patients compared with mild-motor-predominant patients. Men and women showed HRs of 9.9 (CI 2.0–49.5) and 10.8 (CI 1.9–62.8), respectively, for developing HY5 during the observation period (diffuse-malignant vs mild-motor-predominant). Diffuse-malignant patients had an increased risk for walker usage and nursing home living compared to the intermediate group (adjusted HRs of 3.04 CI 1.1–8.1 and 3.14 CI 1.3–7.5, respectively). For women, the diffuse-malignant group’s risk of reaching HY5 was strongly increased compared to the intermediate patients’ risk during our observation period; however, this subgroup was small (Tables 1 and 2, Supplementary Fig. 1).

**Table 2:** Hazard ratios for reaching disease milestones.

Milestone	Unadjusted HR (95% CI)	*p* value	Adjusted HR^a^ (95% CI)	*p* value	Unadjusted HR (95% CI)	*p* value	Adjusted HR^a^ (95% CI)	*p* value
	DM vs MMP				PIGD vs TD			
Walker	2.89 (1.15–7.26)	0.024*	2.81 (1.10–7.15)	0.031*	1.63 (0.74–3.56)	0.224	1.87 (0.85–4.13)	0.119
Nursing home	5.24 (2.25–12.24)	0.000***	3.86 (1.57–9.52)	0.003**	2.36 (0.82–6.80)	0.113	2.03 (0.69–5.95)	0.199
HY5 (women)^b^	16.2 (3.63–72.34)	0.000***	10.79 (1.85–62.81)	0.008**	2.34 (0.49–11.08)	0.283	2.10 (0.43–10.29)	0.361
HY5 (men)^b^	13.01 (2.79–60.72)	0.001***	9.92 (1.99–49.46)	0.005**	4.66 (0.62–35.08)	0.135	5.76 (0.74–45.04)	0.095
Dementia	4.66 (1.49–14.58)	0.008***	4.21 (1.19–14.93)	0.026*	3.41 (0.78–14.87)	0.102	3.32 (0.75–14.68)	0.114
Mortality	4.74 (2.13–10.54)	0.000***	2.67 (1.11–6.43)	0.029*	3.62 (1.1–11.88)	0.034*	2.84 (0.86–9.40)	0.088

	DM vs IM				PIGD vs undetermined			
Walker	2.43 (0.96–6.17)	0.061	3.04 (1.14–8.11)	0.026*	1.37 (0.60–3.13)	0.456	1.73 (0.75–3.99)	0.196
Nursing home	3.54 (1.56–8.05)	0.003**	3.14 (1.31–7.48)	0.01**	1.47 (0.56–3.85)	0.437	1.35 (0.50–3.61)	0.551
HY5 (women)^b^	27.71 (4.43–173.45)	0.000***	63.66 (7.15–567.14)	0.000***	0.85 (0.22–3.25)	0.817	0.83 (0.21–3.30)	0.788
HY5 (men)^b^	2.9 (1.11–7.53)	0.029*	1.49 (0.54–4.07)	0.438	5.13 (0.68–38.63)	0.113	5.31 (0.70–40.50)	0.107
Dementia	2.74 (0.94–7.93)	0.064	2.32 (0.72–7.47)	0.159	2.56 (0.59–11.16)	0.211	2.13 (0.46–9.79)	0.331
Mortality	3.28 (1.51–7.1)	0.003**	2.00 (0.86–4.66)	0.107	2.47 (0.87–7.01)	0.091	2.51 (0.86–7.27)	0.091

	PIGD score only							
Walker	1.14 (0.98–1.33)	0.093	1.15 (0.97–1.36)	0.103				
Nursing home	1.24 (1.10–1.40)	0.000***	1.19 (1.05–1.35)	0.007**				
HY5	1.37 (1.22–1.55)	0.000***	1.34 (1.17–1.52)	0.000***				
Dementia	1.17 (1.03–1.32)	0.017*	1.07 (0.95–1.21)	0.269				
Mortality	1.19 (1.09–1.30)	0.000***	1.11 (1.01–1.22)	0.038*				

Cox regression results for unadjusted and adjusted models for the five outcomes studied.

^a^Adjusted models included age at onset, sex, and duration at baseline investigation.

^b^Results for Hoehn and Yahr 5 outcome showed non-proportional hazards and were therefore analyzed on subgroup level based on sex, see Supplementary Table 1 for numerical results of the test for proportional hazards assumption.

*p value < 0.5; **p value < 0.01; ***p value < 0.001.

*DM* diffuse-malignant motor-nonmotor subtype, *HR* hazard ratio, HY5 Hoehn & Yahr stage 5, *IM* intermediate motor-nonmotor subtype, *MMP* mild-motor predominant motor-nonmotor subtype, *n/a* not available due to one group without events, *PIGD* postural instability and gait disorder, *TD* tremor-dominant motor-phenotype.

The motor-phenotypes showed significantly different risks on log-rank test only for mortality and there were no significant adjusted HRs for any outcome (Table 2). After, ad hoc, removing the tremor part of this system, however, the postural instability and gait disorder (PIGD) scores showed significant HRs after adjustment for death, nursing home, and HY5 milestones.

After sensitivity analysis, removing 28 individuals that had imputed UPDRS scores, motor-phenotypes showed significant adjusted HR for walker usage and PIGD scores HRs remained significant only for the HY5 milestone (Supplementary Table 2). For the motor-nonmotor system, with diffuse-malignant as a reference group, the intermediate group showed similar HRs as in the primary results, but the mild-motor-predominant group retained statistical significance of adjusted HRs only for nursing home living and dementia development (Supplementary Table 2).

### Dementia

Dementia ensued in 27 patients (32.9%) after 12.6 ± 5.9 years of disease (Table 1). Of the 16 surviving patients who had >20 years of disease duration, five (31.3%) had developed dementia; their average onset age was 56.9 ± 12.8 years. Among the patients with older onset age, the proportion of patients with dementia was higher: five of nine (55.6%) with onset age over 70 and two of three (66.7%) with onset age over 75, developed dementia. Male sex, older onset age, and longer disease duration contributed significantly to the risk of developing dementia in Cox regression models (Supplementary Table 3). Hallucinations were present at baseline for 17 of the 27 patients who developed dementia in this study. An *X*^2^ test confirmed dementia development the be different in patients with and without hallucinations at baseline, (*X*^2^ = 20.03 *p* < 0.001, 1 degree of freedom). The risk for developing dementia was seven times higher among patients with hallucinations at baseline compared to those without (Cox regression, unadjusted HR 7.4 CI 3.0–18.1, p < 0.001, adjusted HR 7.1, CI 2.5–19.7 *p* < 0.001; Supplementary Fig. 2, Supplementary Table 3).

### Re-examinations

**Fig. 2:**
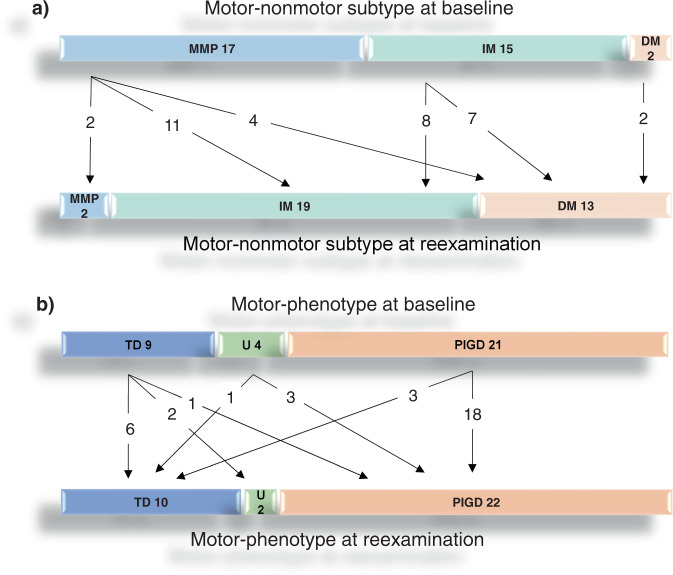
Change in the two classification systems from baseline to the follow-up visit. Baseline classification at the top and classification at reexamination, 8.24 ± 2.0 years later, at the bottom of each section. Numbers indicate individuals that change classification in each system. **a** Motor-nonmotor subtype system, **b** Motor-phenotype system. MMP mild-motor-predominant subtype, IM intermediate subtype, DM diffuse-malignant subtype, TD Tremor-dominant motor-phenotype, U undetermined motor-phenotype, PIGD postural stability and gait disorder motor-phenotype.

Re-examinations were performed in 34 patients, 8.24 ± 2.0 years after the baseline examinations. These patients were then 70.5 ± 8.1 years old and had 15.2 ± 5.3 years of PD duration. Onset age and Unified Parkinson disease rating scale (UPDRS) III score were lower in patients with re-examination compared to those without: 55.3 ± 8.2 vs 62.4 ± 8.8 years, and 15.3 ± 7.3 vs 26.2 ± 11.4 points, respectively. Nonmotor symptoms questionnaire (NMSQ) scores showed a slight difference between re-examined and not re-examined patients, 9.0 ± 4.2 vs 10.4 ± 5.2 points, respectively. Nine women and 27 men had died before re-examination and 53.0% of reexamined patients were men compared to 60.1% at baseline. Hallucinations at baseline were correlated with individual Addenbrooke’s Cognitive Examination Revised (ACER) scores at reexamination (linear regression, unadjusted *B* = −40.3 CI −52.8 to –27.9 *p* < 0.001, adjusted *B* = −37.8 CI −52.0 to −23.5, *p* < 0.001). When both classification systems were re-applied, 29.4% and 64.7% of the re-examined patients changed classification groups in the motor-phenotype and motor-nonmotor systems, respectively (Fig. 2). For these patients, any milestone was only reached if they had PIGD motor-phenotype at baseline or if they had transitioned to PIGD motor-phenotype at re-examination (data not shown).

## Discussion

We used two separate classification systems, motor-phenotype and simplified motor-nonmotor subtypes, to compare risks for major disease milestones in PD. At a mean of 7.9 years of disease duration, we found that the motor-nonmotor system was able to estimate relative risks for using walker, developing dementia, death, nursing home living, and reaching HY5, during the subsequent mean 8.1 years. Motor-phenotypes were unable to successfully stratify risks for reaching the disease milestones during this time, but PIGD score was associated with HY5 development, nursing home living, and death. Furthermore, our simplified motor-nonmotor classification showed high clinical feasibility. Prognostic classification is of importance to enable lifestyle counseling and individualize medical treatment and paramedical care for PD patients and their families.

In the motor-nonmotor system, the diffuse-malignant group showed 2.7–10.8 times the risk of the mild-motor-predominant group for all five disease milestones studied (adjusted HRs; Table 2). Our results support those of previous longitudinal studies, generally substantiating clinical use of the motor-nonmotor system^3,21,23^.

We performed simplifications to two nonmotor subparts in the motor-nonmotor system, introducing NMSQ as nonmotor burden assessment and having experienced hallucinations as a proxy for cognitive assessment. This facilitated data collection and made clinical categorization feasible during one office visit. The proportion of hallucinations were, however, not found to substantially differ between the groups of the original motor-nonmotor system^3^. On the other hand, hallucinations and cognitive decline in PD were clearly linked in our work as well as in other studies^24,25^ and were also found critical for PD subtyping in another study on the same cohort as the original motor-nonmotor system^10^. As hallucinations are more prone to develop with higher age and longer disease duration, the relatively short durations in the original study of the motor-nonmotor system could have obscured a larger contribution of hallucinations in later PD stages. This might, however, speak against using the simplified approach of the motor-nonmotor system soon after the onset of motor symptoms.

The associations found in the present and earlier work on the motor-nonmotor system used different determinations of the nonmotor subparts. The notion that different methods can achieve successful subtyping, could support using different variants of the motor-nonmotor system, with more complex, more precise variants used in research and more easily applied simplifications used in clinical practice.

At the end of this study, the patients had a mean of 16.0 ± 5.4 years of disease duration and 27 patients (32.9%) had then developed dementia. Previous studies on patients with 20 years of PD duration have reported high diversity regarding cognitive outcome; 83% of PD patients developed dementia in one longitudinal study^24^ while the patients in a cross-sectional study showed substantially less cognitive impairment^26^. Two other longitudinal studies evaluated PD development at 10 years of disease and reported 46 and 49% of patients with dementia, respectively^27,28^. Compared with these studies we found a low proportion of patients with dementia, both before and after 20 years of duration, despite using a very broad definition of dementia. We did not aim to investigate reasons for differing dementia incidence, but the relatively low onset age in our study might have influenced this finding as well as possible disinclination to examine or report symptoms of cognitive decline and the fact that we used retrospective chart reviews to determine cognitive decline and dementia.

For the motor-phenotype system we found no significant risk-stratification in adjusted analyses, and conclude that age and duration had a greater impact than the motor-phenotypes at the disease stages we studied, in concurrence with previous findings (Supplementary Table 3)^29,30^. Lack of usefulness of the motor-phenotypes could be due to a confounding effect of disease stage^29–31^. A large proportion, 66.3%, of the present cohort had reached PIGD motor-phenotype at baseline, which likely diminishes the usefulness of this system when it is applied in the middle stage of PD, as in the present study. This is not surprising, since similar ceiling effects have been observed in this system already at 4.5 years of disease duration^20^. Limited usability of motor-phenotypes in mid- and late-stage PD was also indicated for the re-examined patients of the present study where PD milestones were only reached for individuals with PIGD motor-phenotype at inclusion or at re-examination (data not shown), as shown in an earlier study^29^.

We ad hoc used only the PIGD aspect of motor-phenotype, yielding significant HRs of 1.11–1.34 for the death, nursing home, and HY5 milestones. As HRs infer the change in risk per 1 step increase of a covariate, and since PIGD score ranged 0–17 in this cohort, the impact of PIGD score also had a large spread of risks for reaching these milestones. An individual with one SD (2.95) higher PIGD score than others in this cohort, but similar onset age, sex, and duration, had 56.1, 100.3, and 32.5% higher risk for living at a nursing home, developing HY5, and dying during the observation period, respectively. These results support those of another study in which PIGD score but not tremor was associated with negative patient outcome^32^. Significance level of results for death and nursing home milestones changed after sensitivity analysis though, impairing robust interpretation for other milestones than HY5. A strong association between PIGD score and HY5 development could be considered expected because both reflect the severity of axial motor symptoms and balance.

For the motor-nonmotor subtype system, a recent cross-sectional study showed the effects of disease stage and duration on motor-nonmotor subtypes as examined at 5.9 ± 5.4 years of disease duration^23^. In the present work, similar effects were indicated by Cox regression results (Supplementary Table 3), group redistributions at re-examination (Fig. 2), and diverging age of onset and duration in the different motor-nonmotor groups at baseline (Table 1). Nevertheless, these effects did not abolish the simplified motor-nonmotor system’s prognostic capabilities during the observation period, which was longer than other longitudinal studies examining the motor-nonmotor system^3,19–21^, and the present motor-nonmotor groups were also more equally distributed than the motor-phenotypes, both at baseline and after reclassification (Table 1). We conclude that onset age and disease duration substantially affect this system’s risk-stratification capabilities but subvert it to a lesser extent than motor-phenotypes.

Since scales and composite measures of the motor-nonmotor system are valuated relative to the cohort studied, cutoffs must be determined to enable generalizability. We propose that establishing different cutoffs for ranges of onset ages and/or PD durations could compensate for the stage effects observed in the present work and other studies^21,23^.

It has been postulated that the progression rate of PD is more heterogeneous in early–middle than in late disease stages because all patients reach the same neuropathological end-stage^21,33^. In contrast to this concept, we found that the motor-nonmotor system has prognostic value in mid–late PD and may hence convey relevant information to patients, families, and caregivers. PD patients often become confronted with worsening motor control and increasing nonmotor symptoms at this time and will likely benefit from individualized information and care.

Limitations of this study include that four out of five primary outcomes investigated were retrieved from medical records and could be affected by inconsistencies due to different reporters. There might have been selection biases, where participating patients were healthier than average, which might have affected the proportion of patients with dementia. Differences in medication^34^, comorbidities^35^, and education level^36,37^ can affect the outcome and classifications of PD patients. These factors were not adjusted for which could confound results and conclusions made in this study.

Strengths of this study include the relatively long follow-up time, which also solidified the clinical diagnoses, access to the major parts of the patients’ medical records, only three cases lost to follow-up, each assessment performed by the same clinician, and that half of the patients studied were not recruited from a tertiary center but from a geographically defined population.

In summary, we confirmed that a simplified clinical motor-nonmotor subtyping system identifies PD patients at different risks for future disease milestones better than motor-phenotypes. The patients were classified in mid-stage PD with variable and relatively long durations. Both systems showed instability later in the disease course, but our results imply a larger timeframe for the usability of the motor-nonmotor system. Our adaptation of two parameters used in the motor-nonmotor algorithm facilitated classification in the clinical setting. We also confirmed, ad hoc, that when using the motor-phenotype system in mid–late disease the tremor part should be omitted.

## Methods

### Patient cohort

Since 2006, patients with PD were continuously included in a research cohort (PARkinson Lund study; PARLU) consisting of patients with PD living in three municipalities in southern Sweden (population subgroup, 50.6% of the cohort) and patients with familial PD without known genetic cause on testing known to the Department of Neurology at Skåne University Hospital, Lund (hereditary subgroup). For the population subgroup, every resident in three adjacent municipalities (Olofström, Karlshamn, and Sölvesborg) were contacted who had a diagnosis of PD or parkinsonism in registries from all public health care providers in the region between 2006 and 2010, and 76% of those contacted were included in the cohort.

### Patient selection

**Fig. 3:**
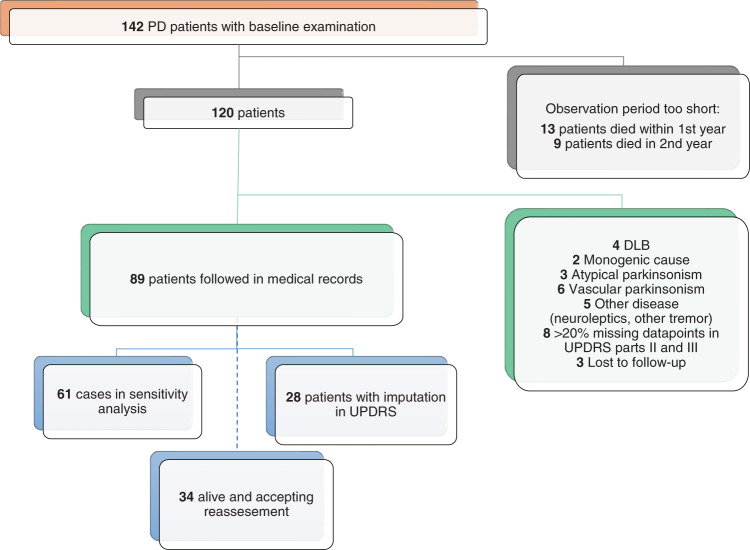
Flow-chart of study design. Atypical parkinsonism included patients with progressive supranuclear palsy and multiple system atrophy. Vascular parkinsonism was defined as lower-body parkinsonism for several years or radiological signs of infarctions in the basal ganglia. *PD* Parkinson’s disease, *DLB* dementia with Lewy bodies, *UPDRS*, unified Parkinson Disease Rating Scale.

Patients within PARLU with PD or PD-dementia and complete baseline visits were selected for this study. Aiming for long-term observation, patients with <2 years of follow-up data were excluded. Patients whose diagnosis had been changed to any other disorder than PD or PD-dementia were excluded, as were two individuals with monogenetic disease (Fig. 3).

### Examinations

Standardized baseline visits were performed from 2007 to 2013. Patients were then interviewed and clinically examined by the same physician (AP) including UPDRS^38^, modified HY-stage^39^, NMSQ^40^, and clinical assessment for other neurological symptoms. The presence of bradykinesia and one of rigidity, tremor, or postural imbalance was confirmed.

In 2017–2018, all surviving patients were invited to a follow-up research visit, including an interview and neurological re-examination by one physician (EYR). The same examination protocol was used with the addition of ACER. Patients used their regular medication on both examinations. We did not measure the doses of dopaminergic therapy because we aimed at evaluating the real-life situation of the patients and because several outcomes of this study were independent of treatment.

### Follow-up data collection from medical records

Time to relevant social and clinical milestones^2^ was extracted from medical records of all individuals: regularly using walker, living in a nursing home, developing HY5, or dementia. This was performed 2018–2019 by one physician (EYR) by searching all medical records from medicine/neurology departments and memory clinics in the southern health care region (regions of Skåne, Halland, and Blekinge) in Sweden, ranging back up to six decades. Medical records from primary health care were acquired when data was missing or inconsistent. All available paper records were scrutinized manually in full length. Electronic records were digitally searched for phrases, words, or parts of words associated with the milestones, including several grammatical forms and common spelling mistakes. Pre-defined criteria for fulfilling disease milestones were used. Patients were considered to have developed dementia when obtaining a diagnosis of dementia not otherwise specified or PD-dementia, being prescribed acetylcholinesterase-inhibitors, or when being repeatedly and clearly described as having dementia in medical records. Disease onset was defined as the first notion of rest tremor or subjective PD motor symptoms. Time at diagnosis was used when no description of onset was available (*n* = 6). To decrease the effect of peri-mortal comorbidities, milestones were ignored if they were only reached within two months before death. Periodicity of follow-up differed between patients and clinics, and to mitigate effects related to the exact timing of the patients’ contact with the medical services (differing interval censoring), all dates when reaching milestones were registered as a calendar year. Dates of birth, death, and end of observation were not standardized but registered as the actual date. The date of death was retrieved in 2019 from the Swedish population register kept by the Swedish Tax Agency, Skatteverket.

### Patient consents and ethical approval

Written informed consent was obtained from all included patients. If the patient was unable or incapable to decide, a close relative was instructed to determine patient consent and to act within the presumed previous intention of the patient. All parts of this study were approved by the Regional Ethics Review Board in Lund.

### Application of classification systems

**Fig. 4:**
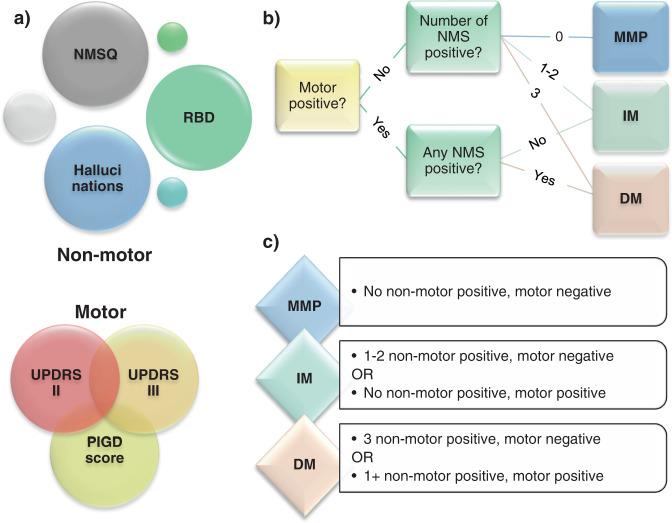
Adaptation of the motor-nonmotor classification. **a** Adaptation of parameters used for clinical assessments. “Motor” was calculated as UPDRSIII + 2.3 × UPDRSII + 21 × PIGD + 55. Motor and NMSQ scores were considered “positive” if above the 75th percentile and “negative” if on or below the 75th percentile. **b** Figure showing grouping process. **c** Figure showing the grouping criteria. *NMSQ* nonmotor symptoms questionnaire, *RBD* REM-sleep behavior disorder, *UPDRS* Unified Parkinson disease rating scale, *PIGD* postural instability, and gait disorder, *MMP* mild-motor-predominant subtype, *IM* intermediate subtype, *DM* diffuse-malignant subtype.

Each patient was classified according to the two classification systems at the baseline examination. The three motor-phenotypes; tremor-dominant, undetermined, and PIGD, were determined by applying cutoffs to a quote between the mean value of selected tremor and postural stability items in UPDRS (PIGD ≤ 1.0 < undetermined < 1.5 ≤ tremor-dominant) as previously described^4^. The three motor-nonmotor subtype groups; mild-motor-predominant, intermediate, and diffuse-malignant, were determined by combining a composite motor score and three nonmotor parameters; a nonmotor rating scale, cognitive assessments, and assessment of REM-sleep behavioral disorder (RBD), similar to the original work^3^. We adapted the nonmotor parameters from the original publication to similar parameters collected in our study (Fig. 4). We used NMSQ instead of Scales for Outcomes in Parkinson’s disease-Autonomic and information provided by the patient and/or caregiver on RBD symptomatology, such as the enactment of dreams, talking, laughing, or screaming while sleeping, replaced the RBD screening questionnaire. As a cognitive marker, we used the occurrence of hallucinations instead of neuropsychological examinations. We considered patients to have had hallucinations if this was indicated in either medical records before examinations or in UPDRS item 2 or NMSQ item 14 at examinations. Thus, RBD and hallucinations had binary (“yes” or “no”) states. NMSQ and composite motor score were continuous rating scales and cutoffs at the 75th percentile of the cohort’s values were used to determine the positive or negative state, as in the original work. We also simplified the composite motor score, derived from averaging individual *z* values of UPDRSII, UPDRSIII, and PIGD subparts of UPDRS as in the original work^3^. We inserted the means and SDs of the present cohort and then mathematically deduced the *z* values to:

$${{{\rm{Composite}}}}\;{{{\rm{motor}}}} = {{{\rm{UPDRSIII}}}} + {{{\rm{UPDRSII}}}} \times 2.3 + {{{\rm{PIGD}}}} \times 21 + 55$$

Of note, the cutoffs for the motor-phenotypes are absolute, whereas the classification in the motor-nonmotor subtypes is per design relative to the distribution of values in the entire cohort studied^3,4^. In our cohort, 75th percentile cutoffs for motor-nonmotor categorization at baseline were 130.4 for composite motor score and 13.5 for NMSQ score. At re-examination, the simplified formula for composite motor score derived at baseline was utilized, but all other aspects of the systems, including cutoffs of the motor-nonmotor subparts (14.8 for NMSQ and 248.7 for composite motor score), was adapted to reexamination data.

### Statistics

Of 141 patients, eight individuals with > 20% of total data points missing in UPDRS parts II and III were excluded (Fig. 3). For 28 patients that were missing ≤ 20% data points (mean ± SD 6.6 ± 5.3%) missing values were imputed with the mean of each patient’s results for the corresponding UPDRS part. We performed a sensitivity analysis without these 28 cases (Supplementary Table 2). A mean of 5.4 individuals (range 0–14) had experienced the milestones before baseline and could not add to Cox regression analyses (Table 1). Linear regressions were performed after the normal distribution of residuals and equality of variances were confirmed. All regression analyses were adjusted for onset age and sex since these factors are known to affect PD severity^41,42^. Adjustments also included disease duration since durations at baseline differed between patients. In each classification system, fulfillment of the five milestones was assessed with Kaplan–Meier survival curves, log-rank tests, and Cox regressions. The group with a worse prognosis in both classification systems was selected as the reference category in Cox regressions. The proportionality of hazards assumption was tested with the cox.zph command of the survival package in R v4.0.2. For all other statistical analyses, SPSS v25.0 was used. In the case of non-proportional hazards, analyses were instead performed in subgroups based on the least contributing covariate (sex), which made hazards proportional (Supplementary Table 1). *P* values < 0.05 were regarded as significant and 95% confidence intervals were consistently used.

### Reporting summary

Further information on research design is available in the Nature Research Reporting Summary linked to this article.

## Supplementary information

Supplementary Information

Reporting Summary

## Data Availability

Statistical protocols or 100% depersonalized original data can be made available to researchers by contacting the corresponding author. According to Swedish law, we are only able to directly share data sets (cohort level/pooled data) that can never be traced back to an individual person. Any data that can be traced back to an individual requires prior written permission by Region Skåne, Sweden.
