# Supplementary material for: Clinical classification systems and long-term outcome in mid- and late-stage Parkinson’s disease
Source: NPJ Parkinsons Dis. 2021 Aug 2;7:66. doi: 10.1038/s41531-021-00208-4 (PMC8329298; doi:10.1038/s41531-021-00208-4)
Supplement: Supplementary file 1 — Supplementary Information [file 41531_2021_208_MOESM1_ESM.pdf]

Ygland Rödström E & Puschmann A, Clinical classification systems and long-term outcome in mid- and late-stage Parkinson’s disease

Supplementary Figure 1 Kaplan-Meier survival curves for men and women

A Motor-nonmotor system

= Mild-motorpredominant   = Intermediate   = Diffuse-malignant

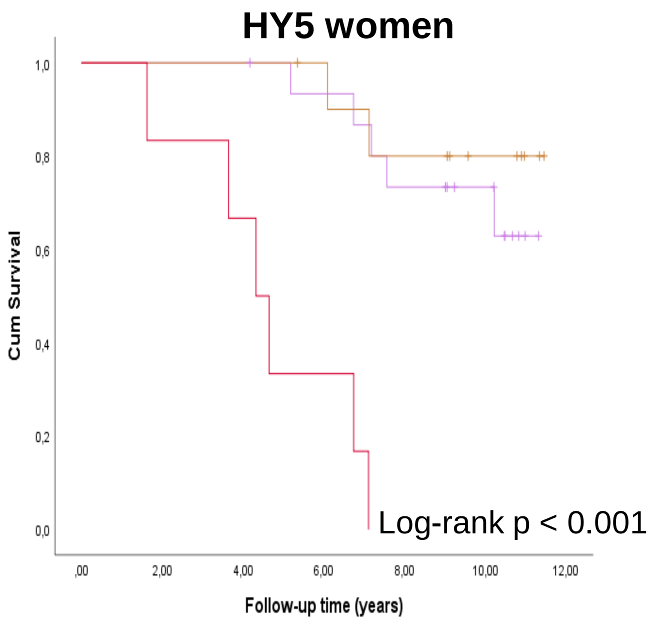

	0	2	4	6	8	10
n	16	16	16	14	11	8
at	11	11	11	10	8	5
risk	6	5	4	2	0	0

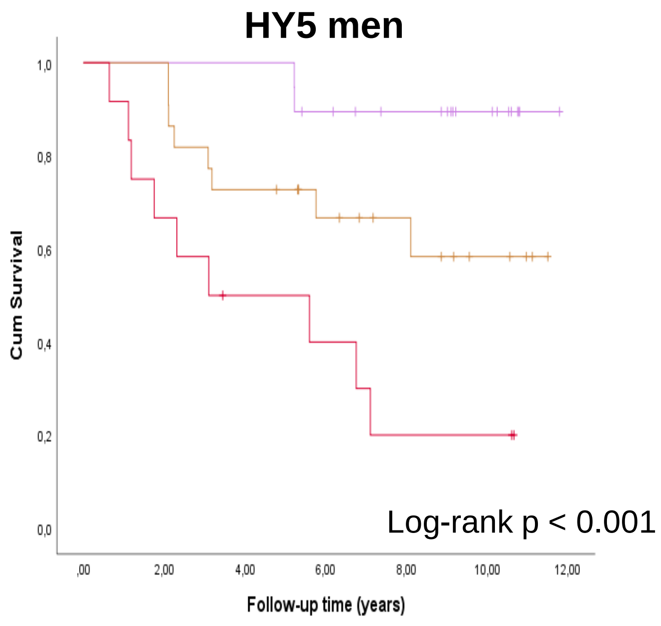

	0	2	4	6	8	10
n	19	19	19	16	13	8
at	22	22	16	11	8	4
risk	12	8	5	4	2	2

B Motor-phenotype system

= Tremor-dominant   = Undetermined   = PIGD

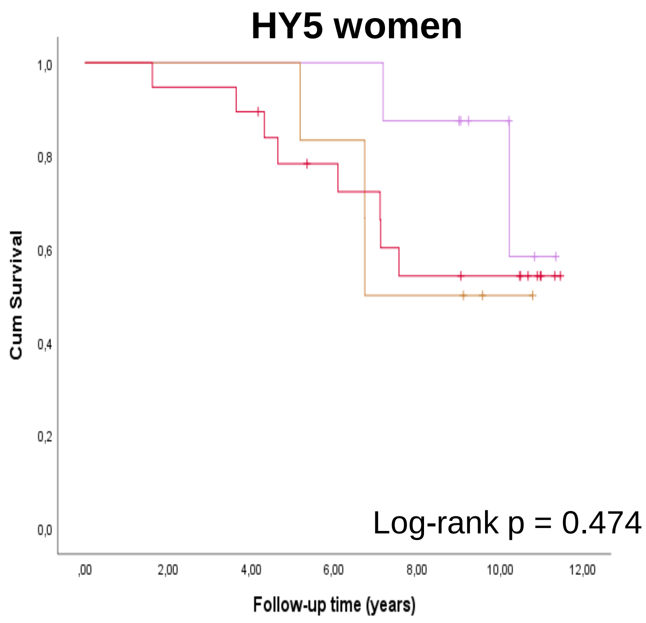

	0	2	4	6	8	10
n	8	8	8	8	7	4
at	6	6	6	5	3	1
risk	19	18	17	13	9	8

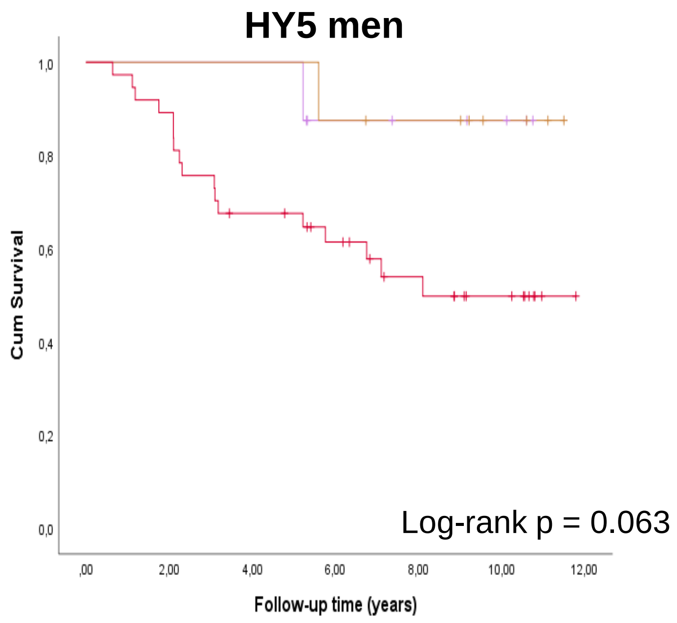

	0	2	4	6	8	10
n	8	8	8	5	4	3
at	8	8	8	7	6	3
risk	37	33	24	19	13	8

Number of cases entering intervals below each graph. **A)** Motor-nonmotor classification system outcomes as indicated; **B)** motor-phenotype system outcomes as indicated. **MMP**, Mild-motor-predominant subtype; **IM**, Intermediate subtype; **DM**, Diffuse-malignant subtype; **TD**, Tremor-dominant motor-phenotype; **U**, Undetermined motor-phenotype; **PIGD**, Postural stability and gait disorder motor-phenotype

Supplementary Figure 2

Ygland Rödström E & Puschmann A, Clinical classification systems and long-term outcome in mid- and late-stage Parkinson's disease

a) Kaplan-Meier survival curves: PIGD score tertiles

= Tertile 1 (blue) = Tertile 2 (green) = Tertile 3 (orange)

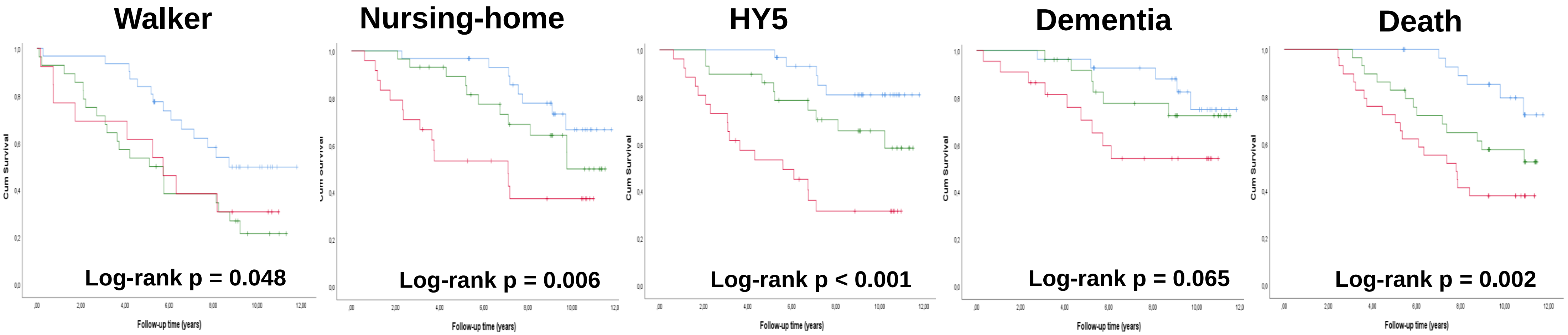

Walker

	0	2	4	6	8	10
n	31	30	29	19	15	7
at	28	24	16	10	10	3
risk	13	9	9	6	5	3

Nursing-home

	0	2	4	6	8	10
n	31	31	30	26	20	10
at	29	29	25	19	15	7
risk	24	19	12	11	7	6

HY5

	0	2	4	6	8	10
n	31	31	31	25	20	12
at	29	29	26	20	15	9
risk	26	21	14	12	7	6

Dementia

	0	2	4	6	8	10
n	27	27	26	21	20	10
at	26	26	22	16	15	9
risk	22	20	15	11	8	6

Death

	0	2	4	6	8	10
n	31	31	31	27	24	14
at	29	29	26	21	18	11
risk	29	29	22	18	12	8

b) Kaplan-Meier survival curve: Hallucinations at baseline

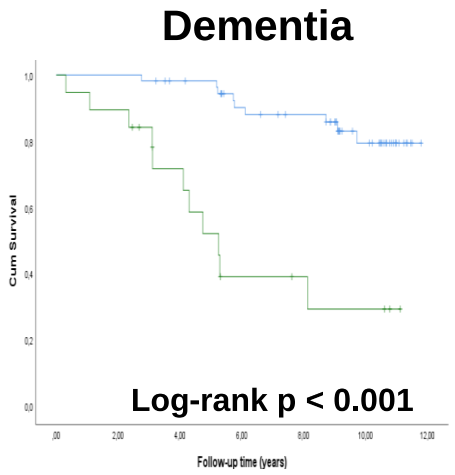

= No hallucination at baseline (blue)

= Hallucinations at baseline (green)

	0	2	4	6	8	10
n at	56	56	52	43	39	22
risk	19	17	11	5	4	3

Number of cases entering intervals below each graph. **MMP**, Mild-motor-predominant subtype; **IM**, Intermediate subtype; **DM**, Diffuse-malignant subtype; **TD**, Tremor-dominant motor-phenotype; **U**, Undetermined motor-phenotype; **PIGD**, Postural stability and gait disorder motor-phenotype

Ygland Rödström E & Puschmann A
Clinical classification systems and long-term outcome in mid- and late-stage Parkinson disease

Supplementary Table 1 Test of proportional hazards

Results from Coxzph command in R (survival package). Adjusted covariates as indicated
*****, non-proportional hazards; **a**, No male patients were TD

Primary data (Allowing imputation)

		Unadjusted		Adjusted
**Walker**	**Subtype**	0.54	SCS-group	0.46
			age	0.64
			Duration	0.52
			Male sex	0.9
			global	0.81
	**Motorphenotype**	0.36	motor	0.64
			age	0.73
			Duration	0.6
			Male sex	0.92
			global	0.89
	**PIGD**	0.11	PIGD	0.14
			age	0.7
			Duration	0.5
			Male sex	0.86
			global	0.52
**NH**	**Subtype**	0.075	SCS-group	0.085
			age	0.45
			Duration	0.656
			Male sex	0.27
			global	0.313
	**Motorphenotype**	0.52	motor	0.66
			age	0.29
			Duration	0.41
			Male sex	0.29
			global	0.79
	**PIGD**	0.25	PIGD	0.43
			age	0.4
			Duration	0.59
			Male sex	0.45
			global	0.85
**HY5**	**Subtype**	0.24	SCS-group	0.146
			age	0.318
			Duration	0.138
			Male sex	0.031*

			global	0,012*
	**Motorphenotype**	0.11	motor	0.275
			age	0.234
			Duration	0.082
			Male sex	0,03*
			global	0,043*
	**PIGD**	0.88	PIGD	0.811
			age	0.483
			Duration	0.226
			Male sex	0,031*
			global	0.063
**HY5 Men**	**Subtype**	0.84	SCS-group	0.95
			age	0.82
			Duration	0.48
			global	0.94
	**Motorphenotype**	0.63	motor	0.8
			age	0.63
			Duration	0.36
			global	0.84
**HY5 Women**	**Subtype**	0.84	SCS-group	0.8
			age	0.28
			Duration	0.46
			global	0.82
	**Motorphenotype**	0.13	motor	0.3
			age	0.17
			Duration	0.17
			global	0.27
**Dementia**	**Subtype**	0.05	SCS-group	0.077
			age	0.23
			Duration	0.792
			Male sex	0.558
			global	0.087
	**Motorphenotype**	0.17	motor	0.24
			age	0.3
			Duration	0.71
			Male sex	0.58
			global	0.29

	**PIGD**	0.57	PIGD	0.9
			age	0.29
			Duration	0.74
			Male sex	0.55
			global	0.49
**Death**	**Subtype**	0.14	SCS-group	0.051
			age	0.471
			Duration	0.743
			Male sex	0.123
			global	0.095
	**Motorphenotype**	0.077	motor	0.16
			age	0.37
			Duration	0.59
			Male sex	0.14
			global	0.27
	**PIGD**	0.1	PIGD	0.14
			age	0.49
			Duration	0.65
			Male sex	0.18
			global	0.44

Sensitivity analysis (Non-imputed cases only)

		Unadjusted		Adjusted
**Walker**	**Subtype**	0.79	SCS-group	0.45
			age	0.43
			Duration	0.78
			Male sex	0.73
			global	0.81
	**Motorphenotype**	0.41	motor	0.59
			age	0.69
			Duration	0.73
			Male sex	0.64
			global	0.85
	**PIGD**	0.14	PIGD	0.39
			age	0.57
			Duration	0.66
			Male sex	0.56
			global	0.81
**NH**	**Subtype**	0.11	SCS-group	0.16

			age	0.93
			Duration	0.79
			Male sex	0.49
			global	0.56
	**Motorphenotype**	0.83	motor	0.95
			age	0.74
			Duration	0.43
			Male sex	0.53
			global	0.95
	**PIGD**	0.095	PIGD	0.2
			age	0.97
			Duration	0.68
			Male sex	0.63
			global	0.76
**HY5**	**Subtype**	0.18	SCS-group	0.073
			age	0.262
			Duration	0.051
			Male sex	0.4
			global	0,019*
	**Motorphenotype**	0,032*	motor	0.129
			age	0.152
			Duration	0,031*
			Male sex	0,027*
			global	0,013*
	**PIGD**	0.64	PIGD	0.952
			age	0.316
			Duration	0.072
			Male sex	0,037*
			global	0.073
**HY5 Men[a]**	**Subtype**	0.85	SCS-group	0.94
			age	0.9
			Duration	0.33
			global	0.63
	**Motorphenotype**	0.77	motor	0.84
			age	0.95
			Duration	0.24
			global	0.68
**HY5 Women**	**Subtype**	0.55	SCS-group	0.35
			age	0.3
			Duration	0.21

			global	0.61
	**Motorphenotype**	0.098	motor	0.214
			age	0.175
			Duration	0.08
			global	0.058
**Dementia**	**Subtype**	0.17	SCS-group	0.22
			age	0.35
			Duration	0.54
			Male sex	0.9
			global	0.51
	**Motorphenotype**	0.27	motor	0.32
			age	0.57
			Duration	0.99
			Male sex	0.99
			global	0.76
	**PIGD**	0.16	PIGD	0.3
			age	0.41
			Duration	0.81
			Male sex	0.91
			global	0.74
**Death**	**Subtype**	0.22	SCS-group	0.027*
			age	0.407
			Duration	0.328
			Male sex	0.334
			global	0.146
	**Motorphenotype**	0,031*	motor	0.092
			age	0.341
			Duration	0.39
			Male sex	0.3
			global	0.244
	**PIGD**	0.05	PIGD	0.091
			age	0.444
			Duration	0.308
			Male sex	0.426
			global	0.412
**Death Men**	**Subtype**	0.55	SCS-group	0.38
			age	0.44
			Duration	0.86
			global	0.66

	**Motorphenotype**	0.23	motor	0.41
			age	0.3
			Duration	0.86
			global	0.54
**Death Women**	**Subtype**	0.94	SCS-group	0.9
			age	0.28
			Duration	0.82
			global	0.56
	**Motorphenotype**	0.14	motor	0.21
			age	0.4
			Duration	0.53
			global	0.2

Supplementary Table 2 Sensitivity analysis

Motor-nonmotor subtype	Unadjusted HR CI	p-value	Adjusted Hr[a] CI	p-value
**DM vs MMP**				
Walker	2,72 (0,95-7,78)	0.063	2,97 (0,97-9,13)	0.058
Nursing-home	4,96 (1,83-13,48)	0.002	3,06 (1,05-8,92)	0.040
HY5-women[b]	10,84 (2,24-52,39)	0.003	4,55 (0,66-31,47)	0.125
HY5-men[b]	18,04 (2,18-148,93)	0.007	8,95 (0,97-82,34)	0.053
Dementia	6,57 (1,54-27,98)	0.011	6,57 (1,31-32,86)	0.022
Death-women[b]	3,37 (0,66-17,1)	0.143	2,64 (0,15-46,09)	0.506
Death-men[b]	6,23 (1,64-23,67)	0.007	2,69 (0,58-12,57)	0.208
**DM vs IM**				
Walker	2,65 (0,91-7,69)	0.074	3,60 (1,14-11,37)	0.029
Nursing-home	3,83 (1,47-10,02)	0.006	3,05 (1,12-8,35)	0.030
HY5-women[b]	45,07 (4,2-484,14)	0.002	34,99 (3,12-391,95)	0.004
HY5-men[b]	3,03 (1,01-9,11)	0.048	1,05 (0,26-4,22)	0.945
Dementia	4,88 (1,30-18,31)	0.019	3,95 (0,92-17,05)	0.066
Death-women[b]	n/a	n/a	n/a	n/a
Death-men[b]	2,71 (0,98-7,49)	0.055	0,84 (0,21-3,32)	0.804

Motor-phenotype	Unadjusted HR CI	p-value	Adjusted Hr[a] CI	p-value
**PIGD vs TD**				
Walker	1,71 (0,58-5,04)	0.329	4,53 (1,32-15,56)	0.016
Nursing-home	2,69 (0,62-11,64)	0.187	3,09 (0,69-13,88)	0.140
HY5-women[b]	1,20 (0,23-6,25)	0.825	2,01 (0,34-11,93)	0.441
HY5-men[b]	n/a	n/a	n/a	n/a
Dementia	2,92 (0,37-23,11)	0.309	3,04 (0,36-25,55)	0.306
Death-women[b]	1,72 (0,19-15,41)	0.629	2,19 (0,22-21,31)	0.500
Death-men[b]	3,51 (0,46-26,64)	0.224	6,83 (0,74-62,76)	0.090
**U vs PIGD**				
Walker	1,12 (0,42-3,01)	0.819	2,62 (0,94-7,34)	0.067
Nursing-home	0,86 (0,32-2,35)	0.773	1,22 (0,43-3,44)	0.706
HY5-women[b]	0,74 (0,17-3,17)	0.688	1,16 (0,23-5,77)	0.856
HY5-men[b]	2,69 (0,35-20,62)	0.342	5,86 (0,73-47,1)	0.097
Dementia	1,16 (0,25-5,39)	0.848	0,71 (0,14-3,70)	0.684
Death-women[b]	0,90 (0,16-4,92)	0.901	1,13 (0,20-6,45)	0.893
Death-men[b]	1,60 (0,36-7,02)	0.533	4,73 (0,98-22,75)	0.053
**PIGD-score**				
Walker	1,21 (1,00 - 1,45)	0.046	1,23 (1,00 - 1,52)	0.051
Nursing-home	1,25 (1,08 - 1,45)	0.003	1,17 (0,98 - 1,39)	0.078
HY5	1,39 (1,20 - 1,61)	0.000	1,32 (1,11 - 1,57)	0.002
Dementia	1,22 (0,99 - 1,48)	0.056	1,26 (0,96 - 1,65)	0.092
Death	1,22 (1,08 - 1,39)	0.002	1,13 (0,95 - 1,35)	0.156

Cox regression results for 61 individuals, excluding 28 individuals with imputated values in UPDRS II or III. **a**, adjusted models included age at onset, sex and duration at baseline investigation; **b**, results showed non-proportional hazards and were therefore analyzed on subgroup level based on sex, see Supplementary Table 1 for numerical results of test for proportional hazards assumption; **n/a**, not applicable due to at least one group with no events. **HR**, Hazard ratio; **HY5**, Hoehn & Yahr stage 5; **TD**, Tremor-dominant motor-phenotype; **U**, Undetermined motor-phenotype; **PIGD**, Postural stability and gait disorder motor-phenotype; **MMP**, Mild-motor-predominant subtype; **IM**, Intermediate subtype; **DM**, Diffuse-malignant subtype

Ygland Rödström E & Puschmann A
Clinical classification systems and long-term outcome in mid- and late-stage Parkinson's disease

Supplementary Table 3 Adjusted cox-regression results for all covariates

Motor-nonmotor group

Walker

	df	p-value	HR (CI)
**Motor-nonmotor group**	2	0.063	
**DM vs MMP**	1	0.031	2,81 (1,10-7,15)
**DM vs IM**	1	0.026	3,04 (1,14-8,11)
**Male sex**	1	0.317	1,39 (0,73-2,64)
**Age at onset**	1	0.000	1,10 (1,06-1,15)
**Duration at baseline**	1	0.000	1,20 (1,10-1,30)

Nursing-home

	df	p-value	HR (CI)
**Motor-nonmotor group**	2	0.006	
**DM vs MMP**	1	0.003	3,86 (1,57-9,52)
**DM vs IM**	1	0.010	3,14 (1,31-7,48)
**Male sex**	1	0.269	1,52 (0,72-3,18)
**Age at onset**	1	0.000	1,14 (1,08-1,20)
**Duration at baseline**	1	0.002	1,15 (1,05-1,26)

Motor-phenotype

Walker

	df	p-value	HR (CI)
**Motor-phenotype group**	2	0.184	
**PIGD vs TD**	1	0.119	1,87 (0,85-4,13)
**PIGD vs U**	1	0.196	1,73 (0,75-3,99)
**Male sex**	1	0.238	1,45 (0,78-2,71)
**Age at onset**	1	0.000	1,10 (1,06-1,14)
**Duration at baseline**	1	0.000	1,19 (1,10-1,28)

Nursing-home

	df	p-value	HR (CI)
**Motor-phenotype group**	2	0.406	
**PIGD vs TD**	1	0.199	2,03 (0,69-5,95)
**PIGD vs U**	1	0.551	1,35 (0,50-3,61)
**Male sex**	1	0.225	1,57 (0,76-3,25)
**Age at onset**	1	0.000	1,13 (1,07-1,19)
**Duration at baseline**	1	0.000	1,18 (1,09-1,29)

PIGD-score only

Walker

	df	p-value	HR (CI)
**PIGD score**	1	0.103	1.1 (1.0 - 1.4)
**Female sex**	1	0.351	0.7 (0.4 - 1.4)
**Age at onset**	1	0.000	1.1 (1.1 - 1.1)
**Duration at baseline**	1	0.000	1.2 (1.1 - 1.3)

Nursing-home

	df	p-value	HR (CI)
**PIGD score**	1	0.007	1.2 (1.1 - 1.4)
**Female sex**	1	0.305	0.7 (0.3 - 1.4)
**Age at onset**	1	0.000	1.1 (1.1 - 1.2)
**Duration at baseline**	1	0.001	1.2 (1.1 - 1.3)

HY5-women[a]

	df	p-value	HR (CI)
**Motor-nonmotor group**	2	0.001	
**DM vs MMP**	1	0.008	10,79 (1,85-62,81)
**DM vs IM**	1	0.000	63,66 (7,15-567,14)
**Male sex**	0		
**Age at onset**	1	0.001	1,25 (1,09-1,43)
**Duration at baseline**	1	0.068	1,16 (0,99-1,37)

HY5-women[a]

	df	p-value	HR (CI)
**Motor-phenotype group**	2	0.577	
**PIGD vs TD**	1	0.361	2,1 (0,43-10,29)
**PIGD vs U**	1	0.788	0,83 (0,21-3,30)
**Male sex**	0		
**Age at onset**	1	0.002	1,14 (1,05-1,23)
**Duration at baseline**	1	0.012	1,15 (1,03-1,29)

HY5

	df	p-value	HR (CI)
**PIGD score**	1	0.000	1.3 (1.2 - 1.5)
**Female sex**	1	0.571	0.8 (0.4 - 1.7)
**Age at onset**	1	0.000	1.2 (1.1 - 1.2)
**Duration at baseline**	1	0.001	1.2 (1.1 - 1.3)

HY5-men[a]

	df	p-value	HR (CI)
**Motor-nonmotor group**	2	0.020	
**DM vs MMP**	1	0.005	9,92 (1,99-49,46)
**DM vs IM**	1	0.438	1,49 (0,54-4,07)
**Male sex**	0		
**Age at onset**	1	0.000	1,21 (1,11-1,33)
**Duration at baseline**	1	0.001	1,29 (1,11-1,51)

HY5-men[a]

	df	p-value	HR (CI)
**Motor-phenotype group**	2	0.080	
**PIGD vs TD**	1	0.095	5,76 (0,74-45,04)
**PIGD vs U**	1	0.107	5,31 (0,70-40,50)
**Male sex**	0		
**Age at onset**	1	0.000	1,22 (1,11-1,33)
**Duration at baseline**	1	0.000	1,34 (1,15-1,56)

Dementia

	df	p-value	HR (CI)
**Motor-nonmotor group**	2	0.083	
**DM vs MMP**	1	0.026	4,21 (1,19-14,93)
**DM vs IM**	1	0.159	2,32 (0,72-7,47)
**Male sex**	1	0.002	7,62 (2,09-27,79)

Dementia

	df	p-value	HR (CI)
**Motor-phenotype group**	2	0.209	
**PIGD vs TD**	1	0.114	3,32 (0,75-14,68)
**PIGD vs U**	1	0.331	2,13 (0,46-9,79)
**Male sex**	1	0.003	7,19 (1,99-25,93)

Dementia

	df	p-value	HR (CI)
**PIGD score**	1	0.269	1.1 (0.9 - 1.2)
**Female sex**	1	0.003	0.1 (0 - 0.5)
**Age at onset**	1	0.000	1.1 (1.1 - 1.2)

	df	p-value	HR (CI)
Age at onset	1	0.000	1,15 (1,07-1,23)
Duration at baseline	1	0.014	1,18 (1,03-1,35)

	df	p-value	HR (CI)
Age at onset	1	0.000	1,14 (1,07-1,22)
Duration at baseline	1	0.002	1,22 (1,08-1,39)

	df	p-value	HR (CI)
Duration at baseline	1	0.009	1.2 (1 - 1.4)

Mortality

	df	p-value	HR (CI)
Motor-nonmotor group	2	0.076	
DM vs MMP	1	0.029	2,67 (1,11-6,43)
DM vs IM	1	0.107	2,00 (0,86-4,66)
Male sex	1	0.000	5,85 (2,41-14,2)
Age at onset	1	0.000	1,17 (1,11-1,23)
Duration at baseline	1	0.001	1,18 (1,07-1,29)

Mortality

	df	p-value	HR (CI)
Motor-phenotype group	2	0.076	
PIGD vs TD	1	0.088	2,84 (0,86-9,40)
PIGD vs U	1	0.091	2,51 (0,86-7,27)
Male sex	1	0.000	6,47 (2,59-16,21)
Age at onset	1	0.000	1,18 (1,12-1,24)
Duration at baseline	1	0.000	1,22 (1,12-1,33)

Mortality

	df	p-value	HR (CI)
PIGD score	1	0.038	1.1 (1 - 1.2)
Female sex	1	0.000	0.2 (0.1 - 0.4)
Age at onset	1	0.000	1.2 (1.1 - 1.2)
Duration at baseline	1	0.000	1.2 (1.1 - 1.3)

Hallucinations

Dementia

	df	p-value	HR (CI)
Hallucinations at baseline, unadjusted[b]	1	0.000	7,38 (3,00-18,14)
**Adjusted**	df	p-value	HR (CI)
Hallucinations at baseline	1	0.000	7,08 (2,54-19,71)
Male sex	1	0.002	9,99 (2,39-41,70)
Age at onset	1	0.000	1,16 (1,07-1,24)
Duration at baseline	1	0.051	1,14 (1,00-1,30)

Cox regression results for the five outcomes studied. Modelst were adjusted for age at onset, sex and duration at baseline investigation unless noted. **a**, results for Hoehn and Yahr 5 outcome showed non-proportional hazards and were therefore analyzed on subgroup level based on sex, see Supplementary Table 1 for numerical results of test for proportional hazards assumption. **b**, unadjusted model. **HR**, Hazard ratio; **HY5**, Hoehn & Yahr stage 5; **TD**, Tremor-dominant motor-phenotype; **U**, Undetermined motor-phenotype; **PIGD**, Postural stability and gait disorder motor-phenotype; **MMP**, Mild-motor-predominant subtype; **IM**, Intermediate subtype; **DM**, Diffuse-malignant subtype
