# Supplementary material for: Clinical classification systems and long-term outcome in mid- and late-stage Parkinson’s disease
Source: NPJ Parkinsons Dis. 2021 Aug 2;7:66. doi: 10.1038/s41531-021-00208-4 (PMC8329298; doi:10.1038/s41531-021-00208-4)
Supplement: Supplementary file 2 — Reporting Summary [file 41531_2021_208_MOESM2_ESM.pdf]

nature research

Corresponding author(s): Emil J Ygland Rödström (emil.ygland@gmail.com)

Last updated by author(s): Jan 8, 2021

Reporting Summary

Nature Research wishes to improve the reproducibility of the work that we publish. This form provides structure for consistency and transparency in reporting. For further information on Nature Research policies, see our Editorial Policies and the Editorial Policy Checklist.

Statistics

For all statistical analyses, confirm that the following items are present in the figure legend, table legend, main text, or Methods section.

n/a	Confirmed	
☒	☐	The exact sample size (n) for each experimental group/condition, given as a discrete number and unit of measurement
☐	☒	A statement on whether measurements were taken from distinct samples or whether the same sample was measured repeatedly
☐	☒	The statistical test(s) used AND whether they are one- or two-sided<br>*Only common tests should be described solely by name; describe more complex techniques in the Methods section.*
☐	☒	A description of all covariates tested
☐	☒	A description of any assumptions or corrections, such as tests of normality and adjustment for multiple comparisons
☐	☒	A full description of the statistical parameters including central tendency (e.g. means) or other basic estimates (e.g. regression coefficient) AND variation (e.g. standard deviation) or associated estimates of uncertainty (e.g. confidence intervals)
☐	☒	For null hypothesis testing, the test statistic (e.g. F, t, r) with confidence intervals, effect sizes, degrees of freedom and P value noted<br>*Give P values as exact values whenever suitable.*
☒	☐	For Bayesian analysis, information on the choice of priors and Markov chain Monte Carlo settings
☒	☐	For hierarchical and complex designs, identification of the appropriate level for tests and full reporting of outcomes
☐	☒	Estimates of effect sizes (e.g. Cohen's d, Pearson's r), indicating how they were calculated

Our web collection on statistics for biologists contains articles on many of the points above.

Software and code

Policy information about availability of computer code

Data collection	No software was used except in the digital search of two medical records system (integrated search in melior, and for NCS Cross, search was in general performed via exportation to adobe acrobat reader or microsoft word).
Data analysis	The proportionality of hazards assumption was tested with the cox.zph command of the survival package in R v4.0.2. For all other statistical analyses SPSS v25.0 was used.

For manuscripts utilizing custom algorithms or software that are central to the research but not yet described in published literature, software must be made available to editors and reviewers. We strongly encourage code deposition in a community repository (e.g. GitHub). See the Nature Research guidelines for submitting code & software for further information.

Data

Policy information about availability of data

All manuscripts must include a data availability statement. This statement should provide the following information, where applicable:

- Accession codes, unique identifiers, or web links for publicly available datasets
- A list of figures that have associated raw data
- A description of any restrictions on data availability

Statistical protocols or 100% depersonalized original data can be made available to researchers on request if complying with the laws of the countries involved.

Field-specific reporting

Please select the one below that is the best fit for your research. If you are not sure, read the appropriate sections before making your selection.

☒ Life sciences ☐ Behavioural & social sciences ☐ Ecological, evolutionary & environmental sciences

For a reference copy of the document with all sections, see nature.com/documents/nr-reporting-summary-flat.pdf

Life sciences study design

All studies must disclose on these points even when the disclosure is negative.

Sample size

We subjectively estimated that the cohort-size could be large enough for the analyses planned without performing any sample-size calculations. The cohort studied was recruited from patients with Parkinson's disease diagnosis living in one of three municipalities in southern Sweden and patients with Parkinson's disease and heredity, known to the Department of Neurology at Skåne University Hospital, Lund, during 2006-2013.

Data exclusions

Pre-determined exclusion criteria were: Patients whose diagnosis had been changed to any other disorder than PD or PD-dementia. Individuals with atypical parkinsonian syndromes, confirmed monogenetic disease, or vascular parkinsonism (defined as lower body parkinsonism for several years or radiological signs of infarctions in the basal ganglia) and others (please see manuscript text and flowchart). Exclusion criterium that was not determined before the start of the study was that patients with less than two years of follow-up data were excluded. This exclusion criteria was added as soon as we realized that several individuals had died in 1-2 years of baseline and that their follow-up did not include enough information to determine whether the studied outcomes were reached or not.

Replication

This was a longitudinal cohort study that reports on outcome in mid-late Parkinson's disease and includes replication of two previously established classification systems.

Randomization

The study did not include randomization since the design of the study was descriptive and observational.

Blinding

Examinator on reexamination was blinded to clinical status and baseline measures.

Reporting for specific materials, systems and methods

We require information from authors about some types of materials, experimental systems and methods used in many studies. Here, indicate whether each material, system or method listed is relevant to your study. If you are not sure if a list item applies to your research, read the appropriate section before selecting a response.

Materials & experimental systems

n/a	Involved in the study	
☒	☐	Antibodies
☒	☐	Eukaryotic cell lines
☒	☐	Palaeontology and archaeology
☒	☐	Animals and other organisms
☐	☒	Human research participants
☐	☒	Clinical data
☒	☐	Dual use research of concern

Methods

n/a	Involved in the study	
☒	☐	ChIP-seq
☒	☐	Flow cytometry
☒	☐	MRI-based neuroimaging

Human research participants

Policy information about studies involving human research participants

Population characteristics

Patients with Parkinson's disease or Parkinson's disease and dementia. Mean (SD) age at symptom onset was 59.7±9.2, Mean (SD) duration at baseline examination was: 7.9±5.3 and 60.7% of the cohort were male.

Recruitment

Every resident in three adjacent municipalities (Olofström, Karlshamn and Sölvesborg) in southern Sweden, were contacted who had a diagnosis of Parkinson's disease or parkinsonism in registries from most health care providers in the region between 2006 and 2010. Individuals with Parkinson's disease that had the disease in the family (at least one 1st or 2nd degree relative or two or more cousins with Parkinson's disease) were also informed and asked to participate if known to the Department of Neurology at Skåne University Hospital, Lund, during 2006-2013.
There might have been selection biases in the inclusion, where participating patients were healthier than average, which might have decreased the proportion of patients with outcomes. Especially the individuals that had developed the more severe outcomes (dementia and Hoehn and Yahr stage 5) could have been willing to participate to a lesser extent.

Ethics oversight

All parts of this study were approved by the Regional Ethics Review Board in Lund, including a separate ethical approval for reexaminations.

Note that full information on the approval of the study protocol must also be provided in the manuscript.

Clinical data

Policy information about clinical studies

All manuscripts should comply with the ICMJE guidelines for publication of clinical research and a completed CONSORT checklist must be included with all submissions.

Clinical trial registration	The study was not registrered
Study protocol	All protocols can be shared on request via any of the authors. Full definitions of outcomes and the search words used in digital search are also attached as a separate file in the submission.
Data collection	Preparations for inclusion was made in 2006. Inclusion and baseline examinations was performed in 2007-2013. Collection of follow-up data through medical records (manual and digital) and reexaminations were performed in 2017-2019. Digital search of medical records was performed using a set of predetermined search-words and all scanned correspondence, examinations etc. and also acquired paper records were viewed manually. More digital search words were in a few instances added to catch spelling-mistakes, aberant wording noticed etc.
Outcomes	(also included in attached file and/or manuscript text)<br>Walker use: Defined as the first time needing walker or wheelchair at most times outdoors or sometimes indoors (or when patient becomes bedridden), and did not clearly improved to not having to use it<br><br>Nursing home living: Defined as living at nursing home at least 25% of the time and not moving back to own home at fultime, any residence with health care staff employed.<br><br>Hoehn and Yahr stage 5: First notion of confinement to wheelchair or bed and the patient cannot walk without personal support afterwards.<br><br>Dementia: Treated with memantine or AcEi, Dementia diagnosis or obvious severe memory impairment that affects daily living, or MMT<23 if had better testresults before and patient or relative note problems related to cognition.<br><br>Death: Extracted from the Swedish population register kept by the Swedish Tax Agency, Skatteverket, in 2019.
